# White matter as a monitoring biomarker for neurodevelopmental disorder intervention studies

**DOI:** 10.1186/s11689-019-9295-8

**Published:** 2019-12-16

**Authors:** Meghan R. Swanson, Heather C. Hazlett

**Affiliations:** 1 School of Behavioral and Brain Sciences, University of Texas at Dallas, GR41, 800 W. Campbell Road, Richardson, TX 75080-3021 USA; 2 Carolina Institute for Developmental Disabilities, Department of Psychiatry, University of North Carolina at Chapel Hill, Chapel Hill, 27599 NC USA

**Keywords:** Autism spectrum disorder, Fragile X syndrome, Brain, White matter, Myelin, Neurodevelopment, Intervention, Clinical trial, Treatment

## Abstract

**Background:**

Early intervention is a valuable tool to support the development of toddlers with neurodevelopmental disorders. With recent research advances in early identification that allow for pre-symptomatic detection of autism in infancy, scientists are looking forward to intervention during infancy. These advances may be supported by the identification of biologically based treatment and outcome measures that are sensitive and dimensional.

**Main body of abstract:**

The purpose of this review is to evaluate white matter neurodevelopment as a monitoring biomarker for early treatment of neurodevelopmental disorders. Fragile X syndrome (FXS) and autism spectrum disorder (ASD) as used as exemplars. White matter has unique neurobiology, including a prolonged period of dynamic development. This developmental pattern may make white matter especially responsive to treatment. White matter develops aberrantly in children with ASD and FXS. Histologic studies in rodents have provided targets for FXS pharmacological intervention. However, pharmaceutical clinical trials in humans failed to garner positive clinical results. In this article, we argue that the use of neurobiological monitoring biomarkers may overcome some of these limitations, as they are objective, not susceptible to placebo effects, and are dimensional in nature.

**Short conclusion:**

As the field moves towards earlier detection and early intervention for neurodevelopmental disorders, we encourage scientists to consider the advantages of using neurobiological features as monitoring biomarkers.

## Background

Research across the last two decades has highlighted how intervention early in life leads to improved outcomes for children with autism spectrum disorder and other neurodevelopmental disorders [1–4]. However, many children with neurodevelopmental disorders do not receive early intervention (e.g., treatment from birth to 3 years of age), because they are not identified and diagnosed until they are 4 years of age [5, 6]. Scientists have invested considerable efforts in lowering the age of identification of ASD. The ultimate goal of this work is to also lower the age of entry into treatment, possibly to the first year of life. While it is likely that infant intervention will result in improved and possibly optimal outcomes, one limitation facing infant interventions is the availability of sensitive and dimensional biologically based treatment and outcome measures. There is emerging research showing that neurobiology can serve as a valid susceptibility/risk biomarker for autism spectrum disorder (ASD) [7–9]; however, there has been far less exploration of neurobiology as a monitoring biomarker.

Monitoring biomarkers are assessed serially over time and can be used to provide evidence of an intervention effect [10]. A key feature of monitoring biomarkers is the focus on change. These biomarkers are often assessed before, during, and after an intervention to determine the beneficial value of the intervention. In therapeutic trials, monitoring biomarkers can be used to measure pharmacodynamic effects and early therapeutic responses. More generally, these biomarkers can increase the interpretability of results and provide credibility to trials. The purpose of this review is to evaluate white matter as a monitoring biomarker for neurodevelopmental disorder treatment trials. We use fragile X syndrome (FXS) and autism spectrum disorder (ASD) as exemplars. This article starts broadly by reviewing key features of white matter development that make it an ideal monitoring biomarker. Next, relevant behavioral, neurobiological, and intervention studies in FXS and ASD are reviewed.

## Main text

### White matter development is experience-dependent and responsive to intervention

To be maximally effective, monitoring biomarkers should be plastic and susceptible to change across the intervening time period. Research on early white matter development in humans is an expanding field, but there is already a growing body of work reporting strong brain-behavior associations during infancy. For example, infants with the greatest change over time in the development of the splenium of the corpus callosum across 6–24 months had superior language at 24 months old when compared to infants with less change in splenium development [11]. Other studies have shown that white matter in typically developing infants is associated with general cognition [12–14], visual orienting [15], and working memory [16].

White matter also changes in response to intervention. For example, in healthy adults, white matter has changed in response to new word learning [17], cognitive training (i.e., participants practiced working memory, episodic memory, and perceptual speed tasks) [18], and training in complex visuomotor skills (i.e., participants learned juggling patterns) [19]. Examination of aphasia patients that participated in extensive intonation-based speech therapy revealed increases in volume and fiber numbers in the arcuate fasciculus post-treatment, demonstrating that white matter remains responsive to treatment after brain damage [20]. White matter may also have potential as a predictive biomarker. In a study of adults with schizophrenia, white matter at baseline predicted gains in attention and executive function post-treatment [21].

There have been fewer studies of how treatment impacts white matter in children. One notable exception found that intensive remedial instruction for school-age children that were poor readers resulted in increased fractional anisotropy (FA) in the left anterior centrum semiovale [22]. While not covered in detail in this review, there is also a body of non-human animal research showing white matter changes in response to the environment [23–26]. Together, this literature highlights the close link between behavior and white matter development, and the plasticity of white matter in response to treatment.

### The first years of life are a time of rapid and dynamic brain growth

**Fig. 1:**
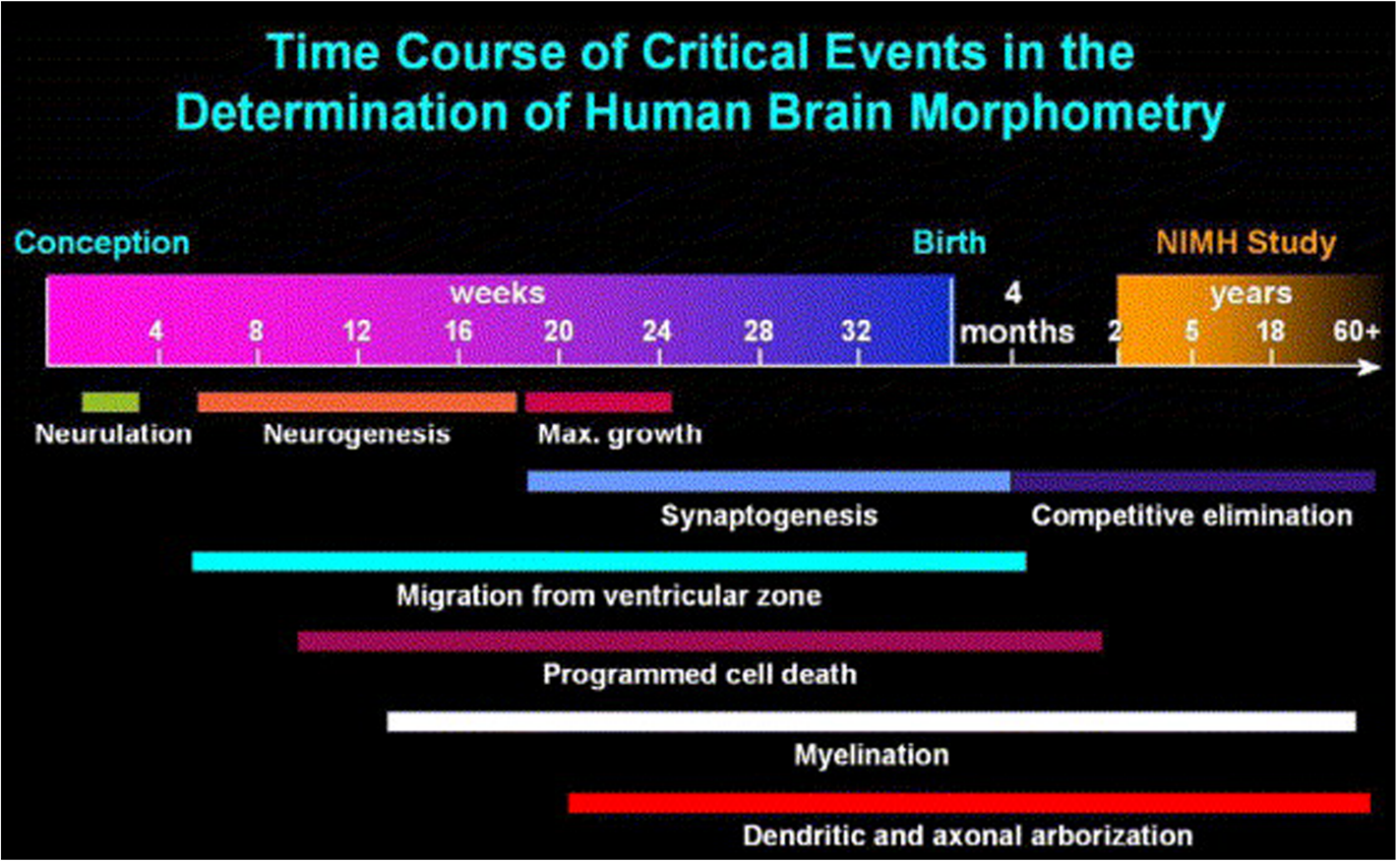
White matter myelination is prolonged process taking place throughout development. Reprinted from [27], with permission from Elsevier

The first few years of life represent a unique time period during development when synaptogenesis, myelination, and pruning are in full swing. During this time the brain is capable of immense growth and plasticity (see Fig. 1). For example, in vivo magnetic resonance imaging (MRI) of human infants has shown that from birth to 1-year brain volume increases 101%. Brain volume growth slows down in the second year with volume increasing an additional 15% [28]. This dynamic growth across the first 2 years of life is largely a result of gray matter growth [29]. In comparison, white matter shows a more prolonged developmental trajectory. The brain’s white matter is mostly made up of myelinated axons that form white matter fiber tracts. These tracts facilitate efficient communication across the brain and allow for fast processing of higher-order cognitive functions. White matter follows a non-linear pattern of development, with maturation mirroring the emergence and refinement of cognitive skills [30]. Anatomically, white matter develops in a posterior to anterior, and inferior to superior manner. The brain stem and internal capsule fiber tracts undergo myelination first and temporal association tracts undergo myelination last [31]. Unlike gray matter which peaks in volume after puberty [32], white matter development is prolonged with volumes increasing into middle age [33, 34]. This prolonged period of dynamic development makes white matter an ideal monitoring biomarker.

### Methodological considerations for measurement of early white matter development

Magnetic resonance imaging of the infant and pediatric brain has the potential to shed new light on the emerging neurobiology of neurodevelopmental disorders, but there are a number of methodological challenges and technical limitations that require attention when collecting and analyzing such data. First, scans acquired during natural sleep without sedation may be difficult to acquire as they require the infant to fall asleep in a foreign environment and remain sleeping during the MRI acquisition. Researchers can provide families with a recording of the MRI machine to desensitize the infant to the loud noise. Proper hearing protection including earplugs and MRI-compatible noise canceling headphones can reduce the likelihood of waking during the scan session (e.g., Optoactive II™, Optoacoustics Ltd., Israel; MiniMuffs, Natus Medical Inc., San Carlos, California, USA). Last, foam pieces can be placed around the infant’s head within the head coil to prevent respiration related motion. Howell and colleagues [35] and Hughes and colleagues [36] review these strategies in detail.

There are a number of technological difficulties related to the image processing of infant MRI that also require careful consideration. Generally speaking, the infant brain has low signal-to-noise ratio (SNR) and is largely unmyelinated. Additionally, changing intensity distributions of white matter, gray matter, and CSF contribute to a difficult environment to development image processing tools for segmenting the infant brain [37]. Taken together, the infant brain is substantially different than the adult brain.

The vast majority of MRI brain templates, or atlases, are based on adult MRI scans. Utilizing these atlases in an infant/pediatric study will result in unavoidable problems such as misclassification of brain tissue, large deformations, and large nonlinear transformations (see [38, 39] for a detailed discussion). Researchers are thus encouraged to utilize either study-specific atlases or age referenced atlases [38–40].

White matter can be measured with a variety of MRI acquisition techniques. Here we focus on diffusion tensor imaging (DTI). DTI is based on tissue water diffusion rates and indirectly measures white matter integrity and connectivity in the brain [41, 42]. Preprocessing steps include correction of motion artifacts (which ideally include automated and visual quality control), brain masking, atlas creation, and co-registration of study DTI images to the atlas. We refer the reader elsewhere for a comprehensive discussion of DTI preprocessing steps [40, 42]. Single tensor tractography, where an anatomically informed fiber track of interest is identified in an atlas and then extracted, is a common approach to quantifying fiber tracks in the infant and pediatric brain. However, the single tensor model is limited in that it over-simplifies anatomy and is susceptible to crossing fibers [43]. Crossing fibers can result in a drop in anisotropy due to poor model fit of the tensor. Single tensor models may also result in false positives, where the tracking algorithms produce tracks that are not anatomically relevant. Researchers can mitigate false positives by creating label maps based on established, anatomically defined regions and cross-referencing the generated tracts with white matter atlases [44]. False negatives are also a limitation of this approach. In this situation, a thinner white matter tract may not be detected at all, or certain portions of a tract may not be captured (e.g., lateral projections of the corpus callosum are rarely observed when using single tensor tractography).

Alternatives to single tensor tractography for diffusion data include voxel-based analyses (VBA) and tract-based spatial statistics (TBSS). The VBA approach is fully automated, time-efficient, and allows for whole brain analysis. VBA is limited in that it assumes perfect registration of the participant data to standard data on a voxel by voxel basis [40, 45]. As such, this approach is not well suited for neurodevelopmental disorders where pathophysiology is likely, nor with infant and pediatric imaging data which require deformable co-registration. TBSS is a specific type of VBA where a white matter skeleton is created, and voxel-wise analyses of the skeleton are performed. This utilization of a white matter skeleton makes TBSS less sensitive to misregistration issues. However, the framework does not provide explicit tract representation and hence information on specific fiber tracts is not available [45]. The whole brain nature of both VBA and TBSS makes these approaches better suited for hypothesis generating studies, and less well suited for a priori hypothesis-driven studies.

Advances in MRI acquisition techniques have led to the advent of high angular resolution diffusion-weighted imaging (HARDI) [46]. HARDI overcomes the issue of crossing fibers by using a larger number of diffusion-weighted gradient directions. This new technique reduces both false positives and false negatives through improved fiber orientation information. It has been argued that HARDI is the most efficient protocol capable of handling crossing fibers [43]. While there are many advantages to HARDI, there is still some uncertainty regarding the ideal acquisition protocols. Additionally, the utilization of high b-values results in low SNR which can make it difficult to perform robust correction for motion artifacts. Ongoing research will undoubtedly resolve these issues.

### Fragile X syndrome and autism spectrum disorder phenotype and genotype

Before examining white matter development in FXS and ASD, we must first provide information on the phenotype and genotype associated with these disorders. FXS is the most commonly inherited cause of intellectual disability, affecting approximately 1 in 7000 males and 1 in 11,000 females [47]. It is an inherited X-linked neurodevelopmental disorder where transcription silencing of a single gene (the *FMR1* gene) results in abnormal expansion of a segment of DNA that contains a repeat of three nucleotides (i.e., CGG triplet repeats in the case of FXS). Typically, this DNA segment is repeated 5 to 40 times; however, in FXS it is repeated more than 200 times. This abnormal expansion silences the fragile X mental retardation protein (FMRP), which in consequence causes nervous system dysfunction. Individuals with 55 to 200 repeats of the CGG segment are said to have the FXS premutation and may experience milder features of the FXS profile. The premutation is associated with both fragile-X-associated primary ovarian insufficiency, a disorder with decreased ovarian function [48], and fragile-X-associated tremor/ataxia syndrome (FXTAS), a late-onset disorder of movement and loss of short-term memory and executive function [49]. The FXS behavioral phenotype includes intellectual disability, social anxiety, attention deficits, and seizures [50–53]. However, intellectual impairments are not uniform, with relative strengths in vocabulary knowledge, and weaknesses in abstract reasoning, attention, short-term memory, and visual-motor coordination.

ASD is a neurodevelopmental disorder with a strong, but complex genetic basis [54]. In families with one child with ASD, empirical evidence suggests that ASD recurrence risk for subsequently-born children may be as high as 19% [55]. Epidemiological population-based estimates put recurrence risk closer to 10% [56]. Children with ASD experience difficulty with communication and interacting with others. They may also have repetitive or stereotyped behaviors and restricted interests. For about 25% of cases, the genetic cause of ASD is known, but no single genetic cause accounts for a substantial amount of total cases [57].

Phenotypically, there is a pre-symptomatic period for children with ASD during the first year of life when the defining behavioral features of ASD have not yet fully manifested. However, this pre-symptomatic period is quite short, with numerous reports of atypical development in core ASD domains such as language and repetitive behavior at 12 months of age [58–62]. Difficulties in other developmental areas have been observed before the first birthday including atypical: motor skills, visual reception [60], eye gaze to social scenes [63], and eye gaze to faces [64]. Research from the Infant Brain Imaging Study (IBIS) Network has demonstrated that in the first year of life infants who go on to have ASD also show atypical neurodevelopment in cortical surface area development [7], functional connectivity [8], white matter development [65], and extra-axial fluid volumes [9]. Some of these pre-symptomatic brain features independently predict later ASD diagnosis with a high degree of accuracy [7, 8]. These recent advances have implications for early ASD treatment, since it is widely acknowledged that early intervention results in improved outcomes for children with ASD [1–3]. It is possible that intervention efficacy could be maximized if it were instantiated during the first year of life, before aberrant brain and behavioral development are increasingly entrenched.

### Atypical early white matter development in NDDs

**Fig. 2:**
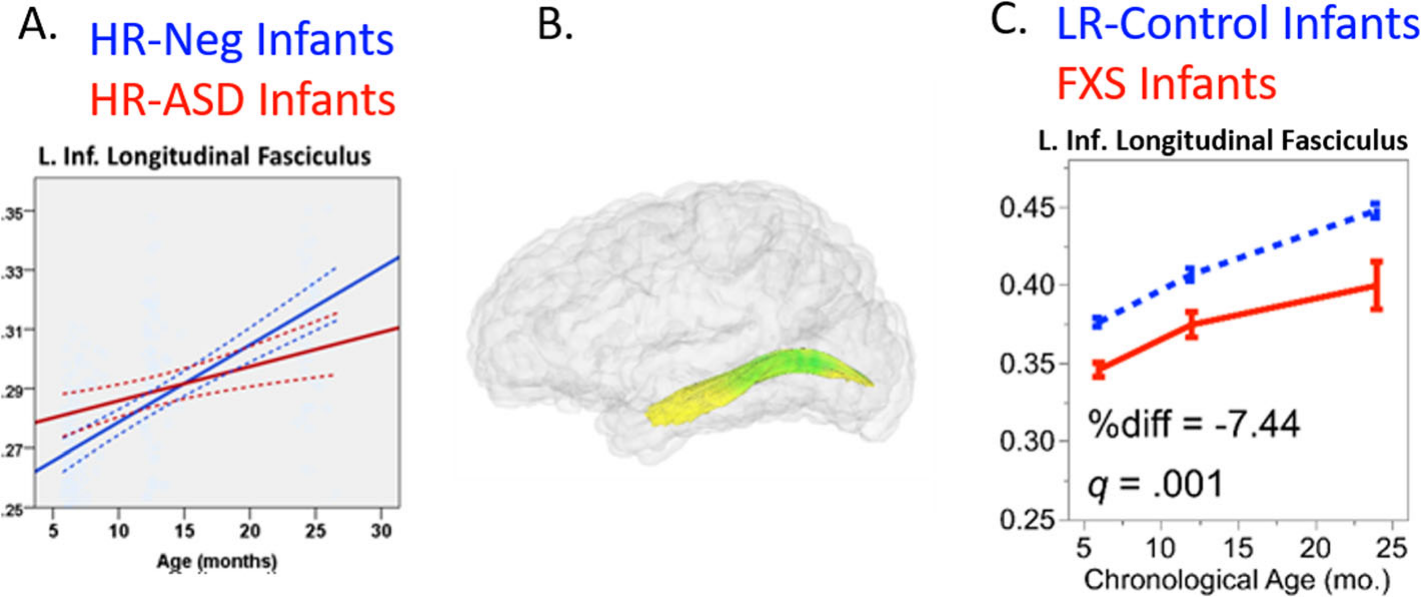
**a** Longitudinal trajectories of FA development in the left inferior longitudinal fasciculus (ILF) in HR-ASD (red) and HR-Neg (blue) infants (reproduced with permission, Wolff et al., 2012). **b** The left ILF in a glass brain. **c** Longitudinal trajectories of FA development in the ILF in infants with FXS (red) and control infants (blue). Panel **a** courtesy of Jason Wolff. Panels **b** and **c** reprinted from [66], with permission from American Medical Association

Atypical development of major white matter fiber tracts is a neurodevelopmental feature of both FXS and ASD. Wolff and colleagues [65] utilized an infant-sibling research design to longitudinally follow infants at high familial risk for ASD from 6 to 24 months of age. Results showed that infants who went on to have ASD themselves (HR-ASD) had higher FA at 6 months, followed by blunted FA development such that by 24 months, they had lower FA values when compared to infants that did not go on to have ASD (HR-Neg) (see Fig. 2a). This pattern of white matter development was widespread with major white matter fiber tracts across the brain showing atypical development. In other work by the same group, Elison and colleagues found that infants that went on to have ASD also showed abnormal visual orienting [15], a foundational skill for early attention that may have cascading effects on joint attention and early language acquisition [67]. Interestingly, only the low-risk control group showed functional coupling between visual orienting latencies and fiber properties of the splenium of the corpus callous, suggesting that the neurocircuitry supporting visual orienting is uniquely disrupted in infants who go on to have ASD. In addition to atypical white matter development in major fiber tracts, infants with ASD may also have neurobiology characterized by white matter network inefficiencies, especially in regions involved in low-level sensory processing [68, 69].

Studies of toddlers and young children with ASD show that atypical white matter development extends past infancy. In a mixed cross-sectional and longitudinal study of young toddlers with ASD (*M* age = 30 months), ASD toddlers entered the study with higher FA in the anterior corpus callosum, but this effect dissipated after 3 to 4 years of age [70]. Similar associations were found for the uncinate fasciculus, arcuate fasciculus, and the inferior frontal-superior tract. However, longitudinal data was only collected on 14 of 61 ASD toddlers so conclusions about trajectories of FA development should be tempered. Cross-sectional studies contribute to the picture of dynamic changes in white matter across the first years of life. As a whole, these studies show higher global FA in ASD when samples include younger toddlers [71–73], and lower global FA when samples are older [74]. These studies highlight white matter as a neurobiological feature that is both dynamic in development and atypical in ASD. However, a definitive prospective and longitudinal study of white matter across infancy into early childhood is needed to fully appreciate the nature of white matter development in ASD.

In the first published report of brain development of infants with FXS, Swanson, Wolff, and colleagues [66] showed that white matter development is atypical by 6 months of age in infants with FXS. In this study, brain scans of infants with FXS and typically developing controls were collected at 6, 12, and 24 months of age. Results indicated that 12 of the 19 major white matter tracts investigated differed by group, with tracts in FXS infants consistently characterized by lower FA over the 6–24 month age interval when compared to control infants (e.g., see Fig. 2c). Fiber tracts connecting subcortical regions (e.g., superior cerebellar peduncles and anterior limb of the internal capsule) and corpus callosum tracts linking primary and premotor cortices were particularly impacted. These findings were the first to substantiate the essential role of FMRP in the early development of white matter connectivity in human infants. The results are in line with other reports showing FMRP is expressed differently across the brain with pronounced expression in subcortical sensory and motor cells [75, 76]. Of particular note, these results are in contrast to those reported in ASD [65, 70, 77], where infants and toddlers with ASD initially show high levels of FA followed by a period of slower development resulting in lower FA values in ASD when compared to controls. These studies contribute to the emerging understanding that FXS and ASD have distinct neural signatures [78–81].

### Early behavioral identification and intervention for ASD and FXS

A common thread across neurodevelopmental disorders is that parent concerns are reported well before the average age of diagnosis. Parents of children with ASD frequently experience delays of 2.5 years between reporting concerns and receiving a diagnosis [82, 83]. Parents of children with FXS experience similar delays in receiving a diagnosis [6, 84]. This timeline is even more prolonged for females with FXS who tend to be less severely affected. There is a new population screening program being implemented in North Carolina that includes voluntary genetic screening for FXS [85]. These programs may reduce drastically lower the age of diagnoses for young children with FXS and make infant intervention feasible.

Despite these frequent delays between first concern and diagnoses, some children with ASD and FXS receive early intervention before the age of three. The advent of early intervention was a watershed moment for the field of autism research. These programs have the overarching goal of providing the best opportunity for optimal development. Best practices for autism early intervention include (1) treatments that include both developmental and behavioral approaches, (2) active involvement of families, (3) consideration of socio-economic and cultural family factors, and (4) practices that consider developmental readiness for learning [86, 87]. Researchers have noted the need for more inclusion of families from diverse backgrounds in early interventions studies, and the need for programs specifically designed for the 0–3 age range [86].

Early intervention efforts to date have shown promise in supporting development in toddlers with ASD, specifically in areas of imitation [88, 89], IQ, and adaptive skills [3, 90, 91] (for a review, see [86]). While fewer studies have shown improvement in the core features of ASD [92], it is widely acknowledged that early intervention results in improved outcomes for children with ASD [1–4]. These positive results may be a harbinger for the potential of infant ASD intervention. Unlike interventions for ASD that have focused on behavioral treatments, FXS interventions have included both pharmacotherapy and behavioral approaches. As such, before examining the relevant literature on early intervention for FXS, we briefly review how rodent models of FXS identified pharmacological targets.

### Rodent models as a tool to identify pharmacological a targets for FXS clinical trials

Non-human animal models of neurodevelopmental disorder have shed important light on the biological basis of these disorders. Researchers have modeled the characteristic gene mutation of FXS and created Fmr1 knock-out mice (for a recent review see [93]). FXS mice are characterized by having altered synaptic plasticity and an overabundance of dendritic spines that appear immature (e.g., spines are long, thin) [94]. These neurodevelopmental changes may be the result of atypical experience-dependent responses at the earliest stages of life. For example, FXS mice exposed to novel sensory input in the second postnatal week did not show the same significant increases in spine density as seen in wild-type mice [95]. However, mice that were exposed to enriched environments for longer periods of time had improved behavioral and morphological features, including more mature spines [96]. These findings suggest that intervention in humans needs to be prolonged and commenced during infancy to be maximally effective.

Several studies of FXS knock-out mice models have reported the recovery of neurological and behavioral symptoms associated with FXS (for a review, see Hagerman et al., [97]). Using induced pluripotent stem cells (iPSC) and CRISPR technology, researchers were able to restore FMRP protein level [98, 99]. Targeting a p21-activated kinase (PAK) inhibitor resulted in reversing the FXS dendritic spine phenotype and also reduced seizures and behavioral abnormalities [100]. In a recent study that used CRISPR-Cas9 to reverse the hypermethylation of CGG expansion, results indicated the rescue of electrophysiological abnormalities in FXS iPSCs [99]. When edited neurons were engrafted into mouse brains, FMR1 reactivation was sustained. It is currently unknown how DNA methylation editing specifically impacts white matter development in the rodent brain. These studies highlight promising advances in identifying therapeutic targets for FXS. However, translating findings from rodent studies to human clinical application has been a challenge, and positive outcomes have been elusive. This topic is explored more fully below.

### Pharmacological intervention trials for humans with FXS

As briefly reviewed earlier, non-human animal research in FXS has resulted in an improved understanding of the pathophysiology of FXS. These research advances led to several drug targets. Generally, drug targets aim to address the excitatory/inhibit neurotransmitter imbalance thought to be present in individuals with FXS. One drug trial tested Mavoglurant, a metabotropic glutamate receptor subtype-5 (mGluR5) antagonist. Results of open label studies of Mavoglurant showed that the drug was tolerated, long-term safety was confirmed, and the FXS patients showed modest improvements in behavioral symptoms [101]. However, randomized, placebo-controlled, double-blind studies failed to report positive effects [102]. These results led Novartis, the manufacturer of Mavoglurant, to discontinue trials of the drug in 2014. Clinical trials of other FXS drugs have likewise failed to show improvements in primary endpoints [103], and have been plagued with methodological weaknesses [104] (for a review, see [105]).

New analyses of eye-tracking data collected during the Mavoglurant trials revealed that patients treated with the drug demonstrated an increase in fixations and overall looking time to the eyes of the stimuli relative to baseline, an effect not seen in the placebo group [106]. FXS individuals in the treatment group also showed greater pupil reactivity to faces relative to controls. Interestingly, there was not a dose-dependent relationship between eye-looking and dose of Mavoglurant, but rather participants who received the smallest dose, 25 mg, showed improved looking time and number of fixations, and participants with the highest dose (100 mg) only showed improved fixation counts. Participants who received the middle dosage of the drug (50 mg) did not show improved looking time or fixations to faces. This study highlights how objective neurobiological measurements can be used to measure treatment effects; however, further research is needed to fully understand these perplexing dose-related results.

## Conclusion

In this article, we explore white matter as a potential monitoring biomarker—a biomarker that is assessed serially over time and can be used to provide evidence of an intervention effect [10]. White matter has unique neurobiology, undergoing a prolonged period of dynamic development and not reaching maturation until middle adulthood [33, 34]. We presented studies showing strong relationships between white matter development and behavior during infancy [11–13, 15, 16], and intervention studies showing that white matter development changes in response to treatment in adults [17–20] and children [22]. We also covered literature showing that white matter development is atypical in both ASD and FXS [65, 66], with findings in FXS supported by histological studies in rodents. While advances in the understanding of FXS pathophysiology led to several candidate drug targets, randomized clinical trials in humans were considered failures [102, 103]. These drug studies were limited by methodological issues, including selecting parent-report measures for primary outcome measures.

The use of neurobiological monitoring biomarkers may overcome some of these limitations, as these biomarkers are objective, not susceptible to placebo effects, and dimensional in nature. Using neurobiological features in such a way is indeed a costly endeavor. However, failed trials due to suboptimal outcomes measures are expensive in and of themselves and may lead to erroneous conclusions. If a quantitative biomarker, such as a metric of white matter pre- and post-treatment, could be utilized in a clinical trial, this could provide an objective and quantifiable measurement of treatment and avoid reliance on qualitative reports.

Reductions in white matter have been identified in another neurogenetic disorder, Angelman syndrome, where significant decreases in white matter development have been observed in the Angelman mouse model [107]. The possibility of implementing a clinical trial, utilizing a metric of white matter to monitor treatment change, could emerge from this work. Patients with Angelman syndrome display significant motor impairments, motor deficits also exist in the mouse model, so it may be informative to examine white matter metrics in motor tracts as a monitoring biomarker for intervention. Optimal treatment windows still need to be identified but work with animal models can help guide clinical trials towards the best developmental periods to target to maximize treatment impact. This work in Angelman syndrome highlights the utility of gaining insights from animal models to guide clinical trial work.

Future studies are needed to carefully evaluate the utility of white matter as a monitoring biomarker. While not the focus of the current work, it is possible neurobiological features could also be used as susceptibility/risk biomarkers (e.g., biomarkers used to indicate the potential risk for developing a disease/condition) and diagnostic biomarkers (e.g., biomarkers used to detect a disease/condition). In summary, as the field moves towards earlier detection and infant intervention for neurodevelopmental disorders, we encourage scientists to consider the advantages of using neurobiological features as monitoring biomarkers.

## Data Availability

Not applicable.
